# Cardiac stem cells: A promising treatment option for heart failure

**DOI:** 10.3892/etm.2012.854

**Published:** 2012-12-05

**Authors:** XIAOHUI ZHAO, LAN HUANG

**Affiliations:** Department of Cardiology, Xinqiao Hospital, Third Military Medical University, Chongqing 400037, P.R. China

**Keywords:** cardiac stem cells, heart failure, cardiomyocyte

## Abstract

Cardiovascular diseases are the most common cause of death in the world. The development of heart failure is mainly due to the loss of cardiomyocytes following myocardial infarction and the absence of endogenous myocardial repair. Numerous studies have focused on cardiac stem cells (CSCs) due to their therapeutic benefit, particularly in the treatment of heart failure. It has previously been demonstrated that CSCs are able to promote the regeneration of cardiomyocytes in animals following myocardial infarction. However, the underlying mechanism(s) remain unclear. This review mainly discusses the cardioprotective effect of CSCs and the effect of CSCs on the function of cardiomyocytes, and compares the efficacies of CSCs from rats, mice and humans, thereby contributing to an improved understanding of CSCs as a promising treatment option for heart failure.

## Contents

1. Introduction
2. New opinion of the heart’s own regenerative potential
3. Markers for the characterization of cardiac stem cells
4. Cardioprotective effect of cardiac stem cells
5. Effect of cardiac stem cells on the function of cardiomyocytes
6. Effect of cardiac stem cells on cardiomyocytes from pressure-loaded hearts
7. Comparison of the efficacy of cardiac stem cells from rats, mice and humans
8. Conclusion

## 1. Introduction

It was previously considered that the regeneration of the heart was impossible to affect on a cellular level and that the heart was a terminally differentiated organ with no potential for regeneration. The general opinion was that cardiomyocytes rapidly proliferate during the embryonic and fetal period but exit the cell cycle soon after birth (1–3). However, cardiac stem cells (CSCs) have been successfully identified in the hearts of rats, mice, dogs and humans (4–9). Thus, the heart’s own regenerative potential opens up a new paradigm in cardiology.

A number of studies have demonstrated that the application of stem cells by injection into the cardiac muscle or coronary arteries as part of bypass surgery or cardiac catheterization procedure increases the contractility of perfused hearts following myocardial infarction (10–15).

Experimental results have suggested that CSCs are able to differentiate into three major cardiac cell populations, including cardiomyocytes, smooth muscle cells and endothelial cells (4). Animal experiments have shown the positive effect of CSCs following a heart attack (9). Thus, CSCs may be the optimal stem cells for the treatment of heart failure. Therefore, this review mainly discusses the cardioprotective effect of CSCs and the effect of CSCs on the function of cardiomyocytes, and compares the efficacies of CSCs from rats, mice and humans.

## 2. New opinion of the heart’s own regenerative potential

It is only in the last 15 years that evidence of mitosis has been observed in hearts with acute and chronic ischemic cardiomyopathy (16–18), idiopathic dilated cardiomyopathy (17) and chronic aortic stenosis (19).

Studies have revealed that mitosis occurred in, on average, 14 of 106 myocytes (17), i.e., it is necessary to investigate ∼350 mm^2^ tissue to identify a single cell undergoing mitosis using a confocal microscope (20). Cell division mainly occurs in mononuclear cells, which account for ∼75% of the cells in the myocardium, and is somewhat rare in binucleated cells (21). Binucleated cells are usually larger than mononuclear cells. The authors observed that human cardiomyocytes >30,000 mm^3^ were no longer able to enter the cell cycle (19).

It has also been reported that young and old cardiomyocytes exist simultaneously in the rat heart (22,23). Cells with stem cell surface antigens and myocardium-specific transcription factors were identified in the myocardium. These cells expressed connexin and cadherin and were thus coupled with mature myocytes via gap junctions (23). Therefore, contrary to previous theories, the heart appears to possess regenerative potential (24).

In addition, chimerism (the presence of genetically different cells in the same organism) has been observed in the human myocardium following heart transplantation (26,27).

Several samples were collected from the atria and ventricles of a male patient who had received a heart from a female donor in 2002 and a Y-chromosome was identified in samples by fluorescence *in situ* hybridization (FISH). In a sample, obtained primarily from the atria, there were more cells with stem cell antigens following transplantation. Of these cells, 12–16% had a Y chromosome (28). These results suggest that circulating stem cells are capable of settling in the heart or that the heart is able to attract stem cells.

This view of the heart’s own regenerative potential opens up novel avenues of therapy for heart failure. There is now moderate hope that the severity of heart failure may be reduced by exogenous or endogenous stem cell therapy.

## 3. Markers for the characterization of cardiac stem cells

**Figure 1.**
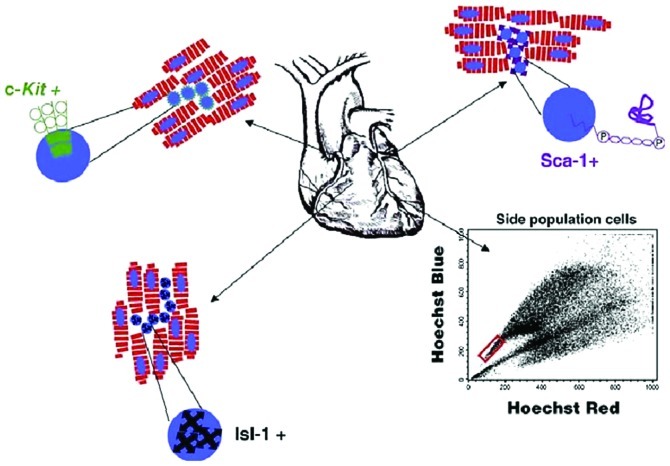
Markers (c-kit, Sca-1 and Isl-1) used for the identification of cardiac stem cells. In addition, the surface markers of ‘side population cells’ express MDR-1.

Stem cells have been detected in a number of tissues, including the blood, skin, central nervous system, liver, gastrointestinal tract and skeletal muscles (29). CSCs have been observed in the hearts of rats, mice, dogs and humans (4–9). CSCs may be identified by various surface antigens. The best known are c-Kit (30), Sca-1 (31), Islet-1 (Isl-1) (32) and multidrug resistance protein-1 (MDR1) (33), as shown in Fig. 1.

Isl-1 is a transcription factor used to identify the cell population which constitutes a substantial proportion of the embryonic heart. Isl-1 expression is downregulated when the cells assume a differentiated phenotype, suggesting that this transcription factor identifies CSCs (34,35).

c-Kit, or stem cell factor receptor, is a protein from the receptor tyrosine kinase (RTK) family and is expressed mostly in hematopoietic stem cells. c-Kit is crucial in the proliferation and differentiation of stem cells (36) and is used as a stem cell marker.

The stem cell antigen Sca-1 is also expressed in hematopoietic stem cells and is commonly used as a stem cell marker (37).

GATA-4 is a cardiac transcription factor and marker of CSCs which is essential in the development of the heart (38). The inhibition of GATA-4 expression results in the inhibition of the terminal differentiation of cardiomyocytes (39).

Atrial natriuretic peptide (ANP) and brain natriuretic peptide (BNP) are considered to be cardiac-specific markers. Their main functions are the reduction of plasma volume and the lowering of blood pressure. ANP and BNP are the main antagonists of the renin-angiotensin-aldosterone system. ANP is a polypeptide synthesized almost exclusively in the cardiomyocytes of the atria and ventricles during hypertrophy of the ventricles. ANP is secreted into the circulating blood in response to atrial stretch, hypoxia or increased plasma osmolarity. BNP is another a polypeptide mainly expressed in the ventricles and atria. The release of BNP occurs particularly in the expansion chambers of the heart due to overload and congestion of the heart. Therefore, ANP and BNP are also defined as cardiac-specific hypertrophic markers (40).

Cardiotrophin-1 is a cytokine and member of the interleukin-6 family. It is expressed mainly in the heart, skeletal muscle, prostate and ovaries and, to a lesser degree, in the lung, kidney, pancreas, thymus, testis and small intestine. Studies have shown that cardiotrophin-1 is detectable only during embryogenesis, primarily in the developing mouse heart (41). Asai *et al*. demonstrated that the human heart secreted cardiotrophin-1 into the peripheral circulation via the coronary sinus (42).

The protein α-actin is located in muscle tissue and is a major component of the contractile apparatus. Thus, α-actin is also studied as a marker for muscle cells and cardiac myocytes.

β-myosin heavy chain (β-MHC) occurs in the adult heart where it forms an integral part of the protein myosin and hence the contractile apparatus. In addition to β-MHC, another isoform of myosin heavy chain, α-MHC, is also expressed in cardiac muscle (43).

The endothelial nitric oxide synthase (eNOS) enzyme belongs to the family of NO synthases. It catalyzes the formation of nitric oxide from the amino acid L-arginine and is involved in the regulation of blood pressure. eNOS was first detected in endothelial cells but was later identified in cardiac myocytes, platelets and brain (44).

## 4. Cardioprotective effect of cardiac stem cells

CSCs may be derived from animal and human hearts. As with the adult stem cells from bone marrow progenitor cells, CSCs are considered to be a potential treatment option for the failing heart. Decisive advantages of CSCs are the possibility of using autologous cells and their ethical acceptability. Several studies have examined their ability to improve cardiac function following myocardial ischemia. Beltrami *et al* isolated CSCs from adult rat hearts and injected them into rat hearts five hours after myocardial infarction, which led to regeneration of the infarcted myocardium (4). Bearzi *et al* reported similar results in the infarcted myocardium after human CSCs were locally injected into immunodeficient mice and immunosuppressed rats (45). In animal models of myocardial infarction, the intramyocardial injection of hepatocyte growth factor (HGF) and insulin-like growth factor 1 (IGF-1) activated the formation of cardiomyocytes and coronary vessels within the infarcted area through their binding to the receptors of the resident CSCs (8,46). The reduction of the infarct size and increase of the left ventricular ejection fraction were observed in a rat model when HGF was administered directly after acute myocardial infarction (47). CSCs and the intramyocardial injection of HGF and IGF-1 resulted in the regeneration of the infarcted area and newly formed myocardial tissue at 20 days after the induction of myocardial infarction in rat hearts (48).

The mechanism underlying the effects described is not yet sufficiently understood. Initially, it was proposed that CSCs or their descendants directly replace the damaged heart tissue, for example*,* following myocardial infarction (4). However, CSCs may cause the release of certain factors by a paracrine mechanism to improve cardiac function and the remodeling process (49). Chimenti *et al* demonstrated that human CSCs secreted vascular endothelial growth factor (VEGF), HGF and IGF-1 after transplantion into immunodeficient mice following acute myocardial infarction. VEGF is also critical in the stem cell-mediated cardioprotective effect (50). In other studies, additional factors have been identified that may be partly responsible for mediating the cardioprotective effects, including sVCAM-1 (51) and interleukin-10 (52). It has also been shown that adult stem cells, particularly mesenchymal stem cells, produce a number of other cytokines, chemokines and growth factors, including matrix metalloproteinases (MMPs), tissue inhibitors of metalloproteinases (TIMPs), monocyte chemoattractant protein-1 (MCP-1), VEGF and interleukins (53). It has been demonstrated that cardiac overexpression of MCP-1 in mice results in a reduction of the infarct area and scar formation and an improvement in left-ventricular dysfunction and remodeling following myocardial infarction (54). Studies of mesenchymal stem cells have also shown that a significant release of VEGF and IGF-1 mediates anti-apoptotic and angiogenic effects and thus exerts a cardioprotective effect (55).

The granulocyte colony-stimulating factor (G-CSF) has been investigated in several studies to determine whether it causes stem cell mobilization and may thus exert a positive impact on cardiac regeneration following acute myocardial infarction. The subcutaneous administration of G-CSF would be less invasive than, for example, the intramyocardial or intracoronary administration of progenitor cells. Despite the successful mobilization of stem cells by G-CSF, neither a favorable effect on the left-ventricular function nor a reduction of the infarct size was observed in patients following myocardial infarction and reperfusion (56).

In spite of all the previous studies, the exact cardioprotective mechanism of CSCs remains poorly understood since it is not possible to distinguish between an improvement in cardiac function and a direct effect on cardiomyocytes in the *in vivo* model. The prevalence of heart failure increases with advancing age, but it is debatable whether the function of stem cells is impaired with increasing age (57).

## 5. Effect of cardiac stem cells on the function of cardiomyocytes

Numerous studies have been conducted to investigate the effect of CSCs on cardiomyocytes but the mechanism underlying the effect remains unknown. The theory that CSCs differentiate into cardiomyocytes directly in the injured heart tissue following myocardial infarction (4) has been increasingly displaced by the hypothesis that CSCs release various factors which affect the heart muscle and surrounding tissue (47,49).

Kretlow *et al* revealed an age-associated impairment of the differentiation potential of stem cells obtained from the bone marrow of mice (58). By contrast, Smith *et al* reported that a significant increase in bone marrow stem cells correlated with increasing age and that the proliferation of hematopoietic stem and progenitor cells did not stop with increasing age in mice (59).

Several studies have already identified several cytokines and growth factors secreted by CSCs. Not all of these factors have confirmed cardioprotective effects and some cytokines even have cardiodepressant effects (60). However, certain factors appear to have a demonstrably positive effect on cardiac function. Other factors secreted by CSCs and other adult and mesenchymal stem cells with notable cardioprotective effects include transforming growth factor beta 1 (TGF-β1), soluble vascular cell adhesion molecule 1 (sVCAM-1), interleukin-10, MCP-1 (54), MMPs and TIMPs. However, the effects of a number of the previously identified factors on the function of cardiomyocytes have never been examined and therefore further studies are required to clarify which factors and mechanisms underlie the observed effects.

## 6. Effect of cardiac stem cells on cardiomyocytes from pressure-loaded hearts

In examining the effect of CSCs on cardiomyocytes obtained from pressure-loaded hearts, it is notable that cardiomyocytes from spontaneously hypertensive rats, as well as those from rats with L-NAME-induced hypertension show no significant changes in their contractile function following a 24-h incubation with CSCs. By contrast, following antihypertensive therapy with hydralazine, the 24-hour incubation with CSCs improved the contractile function at a frequency of 0.5 Hz and this improvement was 4.7±1.8% in the spontaneously hypertensive rats. In the rats with L-NAME-induced hypertension which underwent subsequent antihypertensive treatment, the increase in the contractile function was 10.8±4.0%. The results demonstrate that antihypertensive therapy favors the effect of CSCs in cardiomyocytes from pressure-loaded hearts (61,62).

## 7. Comparison of the efficacy of cardiac stem cells from rats, mice and humans

The effects of CSCs on cardiac function have been investigated in numerous animal models with positive results. A clinical trial in humans is currently underway concerning the therapeutic benefit of progenitor cells on reduced cardiac function following myocardial infarction. The effects of adult stem cells from the bone marrow on cardiac function following myocardial infarction have been evaluated in a number of clinical studies (63–66). The results of these studies demonstrated a decrease in infarct scarring, an improvement of the ejection fraction and a decrease in the left ventricular end-systolic volume. Therefore, it is likely that the effects of CSCs are comparable between animals and humans. For the isolation and culturing of CSCs, tissue samples from the heart rather than the entire heart were collected from humans, while the entire heart was used for mice and rats. This explains why the concentration of human CSCs required was significantly lower than that of rat CSCs to achieve a similar effect. A beneficial effect on the contractile function of cardiomyocytes may also be derived by the use of mouse heart, albeit at higher concentrations than are used for rat and human cells (62).

## 8. Conclusion

This review mainly discusses the cardioprotective effect of CSCs and the effect of CSCs on the function of cardiomyocytes, and compares the efficacy of CSCs from rats, mice and humans, thereby contributing to an improved understanding of CSCs as a promising treatment option for heart failure. It has been demonstrated in animal models that CSCs are able to promote the regeneration of cardiomyocytes following myocardial infarction. This view of the heart’s own regenerative potential opens up novel avenues of therapy for heart failure. There is now moderate hope that the severity of heart failure may be reduced by CSC therapy.
